# Supplementary Treatment for Alleviating Pain and Enhancing Functional Ability in Geriatric Patients with Osteoarthritis

**DOI:** 10.3390/healthcare13020127

**Published:** 2025-01-11

**Authors:** Sorina Maria Aurelian, Corina Oancea, Justin Aurelian, Ruxandra Mihalache, Andreea Iulia Vlădulescu-Trandafir, Alexandru Capisizu, Narcis Chirca, Andreea Zamfirescu

**Affiliations:** 1 Clinic of Geriatrics, Hospital of Chronic Diseases “Sf. Luca”, Faculty of Medicine, University of Medicine and Pharmacy “Carol Davila”, 041915 Bucharest, Romania; sorina.aurelian@umfcd.ro; 2 Department of Physical Medicine and Rehabilitation, Faculty of Medicine, University of Medicine and Pharmacy “Carol Davila”, 050474 Bucharest, Romania; 3 Department of Nursing, Faculty of Midwifery and Nursing, University of Medicine and Pharmacy “Carol Davila”, 050474 Bucharest, Romania; 4 Department of Urology, “Prof Dr Th Burghele” Clinical Hospital, Faculty of Medicine, University of Medicine and Pharmacy “Carol Davila”, 050653 Bucharest, Romania; narcis.chirca@umfcd.ro; 5 Department of Geriatrics, Qualifying Elder Care an Oncopalliative Care, Faculty of Midwifery and Nursing, University of Medicine and Pharmacy “Carol Davila”, 050474 Bucharest, Romania; ruxandra.mihalache@umfcd.ro (R.M.); andreea.zamfirescu@umfcd.ro (A.Z.); 6 Neuromuscular Rehabilitation Clinic Division, Teaching Emergency Hospital “Bagdasar-Arseni”, Faculty of Medicine, University of Medicine and Pharmacy “Carol Davila”, 050474 Bucharest, Romania; andreea-iulia.trandafir@drd.umfcd.ro; 7 Department Physiology II Neurosciences, Faculty of Medicine, University of Medicine and Pharmacy “Carol Davila”, 050474 Bucharest, Romania; alexandru.capisizu@umfcd.ro

**Keywords:** elderly, pain management, osteoarthritis

## Abstract

**Background and Objectives**: A degenerative joint disease that primarily affects elderly individuals, osteoarthritis (OA) causes pain, decreased mobility, and a lower quality of life. Procaine is regarded as a “veteran” medicine due to its extensive clinical use, although it remains a molecule of interest, as researchers are uncovering new biological and pharmacological effects through innovative experimental methods. This study evaluates the efficacy of the “procaine complex”, developed in our country, in alleviating pain and improving functionality in elderly individuals with osteoarthritis of the knee and hip. **Materials and Methods**: We conducted an assessment of a longitudinal short-term study involving 177 patients aged 65 and older, who were randomly divided into two groups. One group received physical therapy and “procaine complex” periarticular injections (n = 101), while the other group received just physical therapy (n = 76). We assessed pain using a visual analog scale (VAS), in addition to functional evaluations using the Lequesne Index, Activities of Daily Living (ADL), and Instrumental ADL (IADL) scores. We evaluated these through a CGA (complex geriatric assessment), the walk test, “Up and Go” test, Mini Mental State (MMSE) and Geriatric Depression Scale (GDS) for cognitive status. We analyzed all the data from this study using PSPP v3 software. **Results**: The procaine complex treatment group exhibited a significant reduction in pain (*p* < 0.001) and improvement in daily activities (*p* < 0.001) relative to the control group. However, there was no notable difference in walking test scores (*p* = 0.171). No substantial detrimental effects were identified. The procaine complex did not surpass physical therapy in reducing depressive disorders, but both groups showed some enhancement in this regard. **Conclusions**: This study demonstrates an innovative approach to pain management by integrating periarticular “procaine complex” injections with physical therapy. This provides elderly individuals experiencing osteoarthritis pain and functional limitations with a secure and efficacious alternative to surgery, or may diminish years of disability.

## 1. Introduction

Osteoarthritis, the most common form of arthritis, is a disease that includes all elements from the synovial joints to the mainly affected components, cartilage and subchondral bones [1]. It is known to be the most common cause of functional failure in elderly individuals [2], usually affecting the hand and large weight-bearing joints, often the knee and the hip [3]. Although the pathogenesis of the disease is not clear, there is molecular evidence regarding the coordinated release of cytokines and inflammatory mediators from elements of synovial joints. Untreated or inadequately managed chronic pain can significantly diminish quality of life, impose functional limitations, and restrict mobility in elderly individuals [4]. In light of the increasing prevalence of OA and the growing need for improved pain management among elderly individuals, it is imperative to explore alternative treatments such as periarticular injections of the procaine complex to alleviate OA-related pain. The progression of OA has been associated with various risk factors including mechanical stress, biochemical abnormalities, genetic predisposition, and metabolic disorders, thus underscoring the importance of investigating novel therapeutic approaches [5,6].

Glucosamine and chondroitin sulfate, either individually or in combination, are widely utilized globally by individuals experiencing osteoarthritis (OA) discomfort. While they are available as over-the-counter dietary supplements in some countries, they require a prescription in others. The prolonged use of nonsteroidal anti-inflammatory drugs (NSAIDs) is associated with an increased risk of cardiovascular issues, gastrointestinal complications, and the progression of osteoarthritis [7]. Managing and preventing these complications incurs substantial costs [8,9]. Recent cohort studies from 2017 onward provide valuable epidemiological insights into OA, with data categorized by affected joint regions and age and different diagnostic criteria, revealing the variability in OA prevalence influenced by demographic factors and geographical locations. For instance, knee OA prevalence ranges from 13.8% in Spain to 35.1% in Korea, while hand OA varies from 7.7% in Spain to 22.4% in the UK. In the U.S., symptomatic hand OA has an incidence rate of 60.5%, and foot and ankle OA prevalence spans from 3.4% in the UK to 22.1% in the U.S. Regarding hip OA, the weighted prevalence for symptomatic cases in Spain is 5.1%, and in Japan, the incidence rate of radiographic hip OA is reported to be 5.6 per 1000 person-years for men and 8.4 per 1000 person-years for women. Together, the results of these research studies demonstrate the high frequency of OA (it is estimated that over 250 million people are affected by this pathology) and its substantial negative effects on health and the economy, making it a major cause of musculoskeletal disability. Additionally, this pathology can complicate the progression of other medical conditions and serves as a significant catalyst for the demand for various medical services worldwide, including geriatrics, rheumatology, rehabilitation medicine, and orthopedics [10,11,12].

The natural aging process impacts the musculoskeletal health of elderly individuals, resulting in joint pain, restrictions in daily tasks, and a decrease in physical activity. Symptoms may encompass cervical, lumbar, or limb pain, as well as rigidity in the upper or lower extremities, and may ultimately result in challenges with ambulation, standing, lifting and transporting heavy items, rising from the ground, dressing, eating, and sleeping. Individuals frequently encounter concomitant fatigue, apprehension, melancholy, anxiety, or profound exhaustion. Worldwide, there are 0.77 billion individuals aged 65 and over, with an estimated 0.5 billion afflicted by a musculoskeletal ailment that would benefit from rehabilitation [13,14].

In the forthcoming decades, we project a doubling of the figures, nearing 0.3 billion elderly individuals who will require substantial orthopedic surgery. These joint, bone, and muscle disorders associated with chronic inflammation limit mobility, may induce weight gain, and interfere with sleep due to pain. Research indicates that musculoskeletal illnesses alone elevate the chance of contracting other non-communicable diseases, including cardiovascular diseases, cancer, diabetes, and mental health disorders, by 17%. The concurrent presence of malnutrition, myosteatosis, and sarcopenia in elderly individuals indicates the potential to improve the physical state of many persons undergoing major orthopedic surgery. Experts acknowledge that these diseases complicate the clinical presentation, presenting substantial hurdles to patients’ recovery, particularly following complex spinal procedures necessitating protracted rehabilitation [15,16].

The procaine complex is a pharmacological formulation created in the mid-20th century in Romania. This chemical, noted chiefly for its local anesthetic characteristics, is a derivative of para-aminobenzoic acid. Procaine, a principal ingredient of GH3, is acknowledged for its analgesic properties, especially in degenerative and age-associated ailments. Its mode of action entails the inhibition of sodium channels in neuronal cells, obstructing the conveyance of pain signals to the brain. In addition to its analgesic characteristics, the procaine complex is linked to anti-aging effects, attributed to its potential in augmenting cellular metabolism and diminishing free radical damage. The procaine complex is clinically utilized in our country for the treatment of arthritis, neuralgia, and several chronic pain disorders, providing alleviation with minimal systemic adverse effects [17,18]. Procaine has shown inhibitory effects on inflammatory mediators like eicosanoids, which are critical in pathological responses to tissue injury and regulatory processes. Moreover, it exhibits anti-inflammatory properties by reducing leukocyte adhesion, granulocyte phagocytosis, and the release of inflammatory mediators and reactive oxygen species. Furthermore, procaine has been shown to enhance oxygen transport within mitochondrial matrices in rat brain studies [19].

Epigenetic mechanisms, particularly deoxyribonucleic acid (DNA) methylation, represent an emerging area of research within the pathogenesis of OA. DNA methylation, which involves adding a methyl group to cytosine–guanine (CpG) dinucleotides, is known to silence genes by blocking transcription factor binding to promoter regions [20,21,22].

Existing research suggests that procaine possesses the capability to reduce CpG island methylation in various cancer types, including hepatoma, breast, pancreatic, and gastric cancer [21,22]. This process has implications for OA, as recent studies suggest that DNA methylation alterations in specific loci affect pathways and molecules associated with OA development. Given these findings, targeting epigenetic changes may present a promising strategy to mitigate OA progression at the molecular level [20,21]. OA’s primary symptoms—joint pain and stiffness—lead to functional disability that significantly reduces patients’ quality of life (QoL) [7]. As it is an irreversible pathology, the main focus is to improve QoL, defined as an individual’s subjective evaluation of their health status based on their own personal and cultural beliefs, along with their goals, expectations, concerns, and standards [23,24]. Knee and hip OA are particularly prevalent and debilitating, and severely restrict the ability to perform essential tasks and sustain an active lifestyle [12].

This controversial gerontoprotector, developed by Ana Aslan in 1953, contains the procaine component, first synthesized by Alfred Einhorn in 1905, and its metabolism involves two compounds: diethylaminoethanolic acid (DEAE) and para-aminobenzoic acid (PAB). Procaine action takes place at the cellular level where it stabilizes cell membranes, reduces the formation and accumulation of free radicals and inhibits the release of lysosomal enzymes [18]. GH3 is a procaine complex, featuring the acetylcholine precursor DEAE, which augments cerebral catecholamine activity, yielding favorable antiparkinsonian effects, and suppresses monoamine oxidase (MAO), thereby eliciting antidepressant effects [25,26]. Another metabolic compound, PAB, inhibits the solubilization of precollagen. According to recent research, proteolytic enzymes have the ability to increase the body’s self-healing mechanisms by promoting and accelerating inflammatory and immunological responses. Additionally, they show a decreased incidence of treatment-emergent adverse events and a good safety profile [27]. Its use as an adjuvant in the treatment of chronic degenerative rheumatism has led to pain control in approximately 92% of patients and to an increase in joint mobility in 50% of these. Research on procaine’s impact on lifespan is limited. An experimental investigation by Aslan et al. (1965) using 1840 rats indicated that treated animals had a lifespan 18–21% longer than that of the control group injected with saline solution [28].

This study seeks to address the deficiency in current treatments for elderly patients with osteoarthritis and chronic pain, specifically the lack of therapies that provide effective pain relief with minimal side effects, while simultaneously enhancing joint health and overall quality of life. Contemporary therapies, such NSAIDs and opioids, frequently have considerable hazards, such as gastrointestinal, cardiovascular, or dependency-related complications, which are especially concerning in elderly populations. This primary study aims to investigate through innovative or integrative methods the efficacy of the “procaine complex”, which specifically targets pain mechanisms, mitigates systemic hazards, and tackles the complex character of osteoarthritis in elderly people. The secondary aim of the study is to integrate pain management using a procaine complex as a complementary therapy into elderly rehabilitation physical programs.

## 2. Materials and Methods

A randomized, interventional, longitudinal for short time clinical trial was conducted to assess the efficacy of a procaine complex in reducing pain and enhancing functional abilities in elderly patients with knee and/or hip OA. This study design was deemed most suitable due to its ability to analyze a well-defined population within a relatively short timeframe.

Participants were assured that their privacy would be respected and that their responses would be analyzed anonymously. Additionally, a limited set of socio-demographic data, including age, gender, and place of residence, was collected.

### 2.1. Study Design

The study comprised 177 patients who were enrolled between February 2023 and July 2024 in the Geriatrics and Gerontology Clinic from “Sf. Luca” Hospital Bucharest, Romania. The study was conducted in accordance with the Declaration of Helsinki and approved by the Ethics Commission of the “Sf. Luca” Chronic Disease Hospital (protocol code 03, date of approval 9 January 2024) and enrolled in ClinicalTrial.gov with ID NCT06717724. All patients provided written informed consent and were hospitalized patients.

#### 2.1.1. Inclusion Criteria

Eligible patients were men and women over 65 years old who had symptomatic osteoarthritis of the knee and/or hip as defined by the criteria of the American College of Rheumatology [29]. The diagnosis of hip OA occurred when at least two of the following criteria were present: the patient experienced pain in the hip internal rotation, morning stiffness for at least 60 min, radiographic signs of osteophytes (bone spurs) on the femoral head or acetabulum, joint space narrowing on imaging (especially superior, axial, or medial), and an ESR (erythrocyte sedimentation rate) of less than 20 mm/h. The diagnosis of knee osteoarthritis was confirmed based on clinical and/or radiological features. At least one of the following symptoms accompanied knee pain: age over 50 years, morning stiffness lasting less than 30 min, crepitus, and the presence of osteophytes [29,30].

#### 2.1.2. Exclusion Criteria

The study excluded 15 individuals who had Alzheimer’s Disease, severe depression, mental illness, cancer of any type, history of any severe disease diagnosis (including complicated diabetes with polyneuropathy, cardiovascular disease such as instable angina, myasthenia gravis, etc.). We removed individuals who had undergone surgical procedures in the last year and those who had received NSAID medication in the past month. We also excluded 4 individuals who were eligible but refused to join the study, so there was a total of 203 participants who took part in this study at the beginning.

#### 2.1.3. Groups

**Figure 1:**
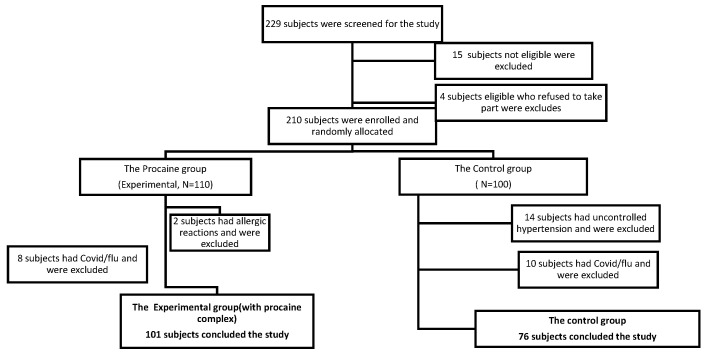
Study flow chart demonstrating the selection and treatment process of involved patients. The selected patients were subject to two examination checkpoints: initially on the first day of hospitalization (T0) and after 10 days of therapy (T1).

Allocation was generated automatically using numerical tables; the 210 participants (Figure 1) who satisfied all eligibility requirements supplied written, informed consent prior to the trial and were randomly assigned to the experimental group (EG, n = 110) or the control group (CG, n = 100). In the experimental group, 2 patients presented allergic reactions to the procaine complex, which led to stopping treatment and exclusion from the study, and 8 patients had COVID-19 or flu (EG, n = 101). In the control group, 14 patients had uncontrolled hypertension and could not undergo physical therapy, and 10 had COVID-19; they were also excluded (Control, n = 76). The primary outcome was the patients’ global assessment of pain in the affected joint, measured with VAS. Other outcome measurements included the Lequesne Functional Index, the ADL and IADL index, demographic characteristics, a cognitive assessment through the Geriatric Depression Scale-15 (GDS-15), and physical performance assessment through the gait speed test and “Up and Go” test.

The scores were assessed on the first hospitalized day (T0) and on the 10th day (T1) following the intervention. The determination of the sample size was influenced by limitations in the research period and available research funds.

### 2.2. Intervention with Procaine Complex

Each participant in the experimental group received a periarticular injectable procaine complex solution (5 mL contains 100 mg procaine hydrochloride) for 10 days. All patients were treated using the sterile injection technique. Participants in the EG (procaine group) did not perform physical therapy daily. Before treatment with the procaine complex, the patients’ sensitivity to procaine hydrochloride was tested via the subcutaneous administration of 0.5 mL injectable, and 2 patients had allergic reactions. The presence of any allergic reaction represents a contraindication to treatment. In the absence of sensibility to the tested substance, the procaine complex was administered.

#### Technique

The more painful and less functional knee was selected for the procedure. The injections were performed with the knee in a flexed position. Antisepsis was administered by applying gauze soaked in 70° GL alcohol, using circular, centrifugal movements three times in succession. The point of entrance of the needle was the femorotibial periarticular interline, 1.5 cm below the apex of the patella to avoid the puncture of the Hoffa’s fat pad [31]. Before injecting, aspiration of the syringe was performed to avoid joint effusion that might be present and to ensure that the needle was not inside a blood vessel. Periarticular injections are well tolerated and safe. Careful injection technique enhances the effectiveness of pain control. This procedure was repeated once daily for 10 consecutive days.

### 2.3. Evaluation Tools

#### 2.3.1. Visual Analog Scale (VAS)

VAS is one of the most commonly used instruments to measure pain in the general population as it is considered the most sensitive, reproducible, and simple pain scale. It is a 10 cm line with anchors at both extremities, with the words “without pain” at one end and “unbearable pain” at the other end. The patient is required to mark a point indicating their pain and a 0–100 mm ruler is used to quantify the measure [32].

#### 2.3.2. Lequesne Index

The Lequesne Index comprises 10 specific questions for patients with knee osteoarthritis: 5 related to pain or discomfort, 1 for maximum distance walked, and 4 regarding daily life activities. The score varies between 0 and 24 points and the higher the score, the worse the pain and function [33].

#### 2.3.3. Activities of Daily Living (ADL)

The Activities of Daily Living are a series of basic activities performed by individuals on a daily basis necessary for independent living at home or in the community. There are many variations on the definition of the activities of daily living, but most organizations agree there are 6 basic categories: personal hygiene, dressing, eating, continence, mobility, and rest. Whether or not an individual is capable of performing these activities on their own or whether they rely on a family caregiver for assistance to perform them serve as comparative measures of their independence. The score varies between 0 and 6 points and the lowest score represents dependence status [34,35].

#### 2.3.4. Instrumental Activities of Daily Living (IADL)

IADL assessments evaluate patients’ ability to manage money, shop, cook, take medication, and use transportation for social integration. The total score ranges from 0 (poorly functioning, dependent) to 8 (high-functioning, independent) [36].

#### 2.3.5. The Geriatric Depression Scale-15

The GDS-15 is a 15-item variation of the GDS with solid reliability, consisting of 15 dichotomous questions (yes or no). Scores range from 0 to 15. A score of 6 or higher, out of a maximum of 15 points, is regarded as a potential indicator of depression [37]. We used the 4 m walk test and the Up-and-Go test to measure physical activity [38].

### 2.4. Physical Therapy

Patients had one exercise session per day in the hospital physical rehabilitation department. Exercise therapies are typically conducted under the supervision of physiotherapists at our hospital. The exercise program, developed through clinical practice and consultation with a rehabilitation physician, were intended to enhance lower-limb muscle strength and balance, alleviate pain, and diminish knee/hip stiffness. Each exercise session included isometric contractions of the quadriceps, a seated knee flexion stretch, seated knee extensions, mini squats (chair-assisted), and stationary bike. Patients were advised to follow 2–3 sets of 10 repetitions for a total workout time of 30–40 min daily, for a duration of 10 days. Not all patients engaged in an identical workout regimen; the physiotherapists devised individualized regimens for the patients, tailored to their physical capabilities and knee complaints.

The diagnoses were grouped as follows: POA = gonarthrosis, coxarthrosis, and osteoarthritis of the peripheral joints; SOA = spine osteoarthritis; SPOA = both central (spine) and peripheral joints affected by osteoarthritis; HD = spinal disc herniation and sciatica; Osteoporosis; and Trauma (fracture).

### 2.5. Statistical Analysis

All parameters were compared between the experimental group of patients who received the procaine complex (EG) and the control group of patients who received only physical therapy (CG). All parameters were compared using the Chi-square test for nominal and categorical variables (gender, residence, diagnosis, and age categories) and the independent-sample *t*-test for numerical variables (age). Normality tests were conducted on the study variables, including graphical methods such as histograms for age. Within-group analyses were performed using the ANOVA test to compare the groups’ outcomes before and after treatment. All statistical analyses were performed using PSPP v3 software.

### 2.6. Ethical Considerations

This study and the informed consent obtained approval from the ethics research committee of a public third-level hospital, “Sf. Luca” Chronic Disease Hospital (protocol code 03, date of approval 9 January 2023), and by ClinicalTrial.gov ID NCT06717724. This study complied with current legislation and was in accordance with the Declaration of Helsinki. All subjects agreed to participate in this study prior to enrollment.

## 3. Results

One hundred seventy-seven patients participated in the study. This group of older patients experienced at least one of the following symptoms/complaints: vertebral pain, memory decline, insomnia, and pain in the knees or hips.

One hundred and one patients received the procaine complex, with a mean age of 70.10 ± 8.53 years. Seventy-six patients received physical therapy (PT), with a mean age of 71.32 ± 9.33 years; the difference was not statistically significant (NS) between the two groups. The difference was not statistically significant (NS) between the two groups for gender either, but it was statistically significant regarding residence: 121 (68.75%) patients from urban areas vs. 55 (31.25%) patients from rural areas; *p* < 0.001. In our study group, we discovered that 50% of the female patients had gonarthrosis. Furthermore, regardless of gender, 22% of those aged 66–74 y developed gonarthrosis, the highest frequency of any age group (Table 1).

The Lequesne Algofunctional Index (LAI) is a questionnaire consisting of 10 interview-based questions, formulated as a cohesive instrument. The ten factors are categorized into three sections: ‘pain or discomfort’ (P/D), ‘maximum distance walked’ (MDW), and ‘activities of daily living’ (ADL). We assessed the pain using both the visual analog scale and the LAI section. The mean values for the analyzed parameters at different time points, T0 and T1, for the two groups of patients, who received either the procaine complex or physical therapy, are presented below (Table 2).

In the group administered the procaine complex (CG), scores demonstrated improvement by the conclusion of hospitalization (at T1 compared to T0), with the exception of gait speed and the depression scale, as detailed below:

* Visual analog scale improvement = VAS T0–T1 (*p* < 0.001);
* Pain or discomfort (P/D) improvement = P/D T0–T1 (*p* = 0.001);
* Maximum distance walked improvement = MDW T0–T1 (NS);
* Less difficulty in performing daily activities = ADL T0–T1 (*p* < 0.001);
* Functional Lequesne Index improvement = LAI T0–T1 (*p* < 0.001);
* Geriatric Depression Scale improvement = GDS T0–T1 (*p* = 0.078).

Interestingly, the data suggested that PT may be more effective than the procaine complex in reducing symptoms of depression (Table 2). After 10 days of treatment, two-group comparison analysis showed significant improvement in VAS, daily activities, and Lequesne Index scores among the procaine complex and the control group.

Among all the parameters, only VAS revealed substantial variations across distinct diagnoses and age groups among the measured parameters (Table 3 and Table 4).

The examined group was categorized into four age groups: the first group comprises individuals aged 65–69 years (adults), the second group includes those aged 70–74 (elderly), the third group encompasses ages 75–84 (very old), and the fourth group consists of individuals over 85 years (oldest old). An analysis of parameter evolution over time is presented in Table 4.

Of all the indicators, VAS score was mostly improved in those with POA osteoarthritis and in the second category (elderly). Additionally, it appears that following the intervention, the fourth category (oldest old) may have the greatest potential for improvement in their walking ability compared to the other categories.

**Figure 2:**
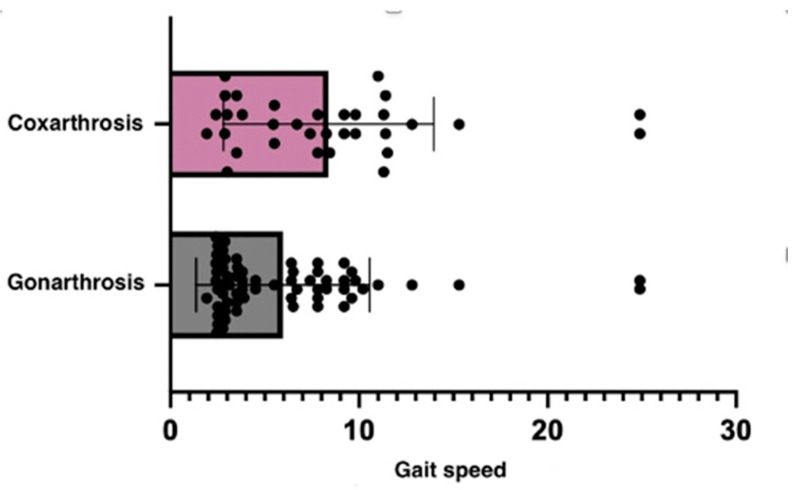
Walking speed distribution among gonarthrosis and coxarthrosis patient groups.

The VAS of pain was assessed in T0 and T1 in the experimental group of patients with gonarthrosis, and statistical analysis revealed significant findings (*p* = 0.0119). The actual change in pain ratings varied from −1.329 cm to −0.1714 cm, according to the 95% confidence interval, indicating that the therapy used had a significant effect in reducing pain in gonarthrosis patients, resulting in an improvement in their clinical condition (Figure 2).

These results suggest that patients with coxarthrosis may experience more severe mobility and balance problems than those with gonarthrosis. The Up and Go test is more impacted in the coxarthrosis group (Table 5). Since the hip joint is in charge of a wider range of motions and activities, its impairment can significantly determine a patient’s mobility and equilibrium.

The study showed that there was no statistically significant variance in the improvement in parameters between coxarthrosis and gonarthrosis.

## 4. Discussion

### 4.1. The Main Findings of the Study

In the analyzed cohort, the majority of patients with gonarthrosis were female and resided in metropolitan areas (*p* < 0.001). Urbanization restricted their physical exercise. The cohort administered the procaine complex (CG) exhibited enhancements in scores by the conclusion of hospitalization (at T1 relative to T0), including VAS (*p* < 0.001), pain or discomfort (P/D) amelioration (*p* = 0.001), reduced difficulty in executing everyday activities, and improvement in the Lequesne Functional Index. Older adults have limitations in their daily activities and performance. In our study, we examined the possibilities of increasing their well-being in terms of relieving pain or depression or augmenting mobility through another intervention besides oral NSAIDs or neuropathic drugs [39,40].

### 4.2. Comparing Findings with Those of Alternative Research

Osteoarthrosis is the most prevalent joint disease and is the main cause of functional incapacity among elderly people [41]. Knee OA is the fourth most common cause of health problems among elderly women [42], and in our study, we found a 52% prevalence.

Our results are also corroborated by those of Giombini et al., who used oxygen–ozone for 23 patients with knee OA and found it effective in relieving pain and improving function and quality of life [43]. In spite of the randomization table being generated by a computer program, there were more patients in the ozone group than in the placebo group. Physical function and physical activity have a well-established direct association that improves health outcomes for older persons [44]. The procaine complex, as a gerovital gerontoprotector, was found to improve pain and mobility, possibly linked to the anti-inflammatory effects reported by former studies [18]. These improvements were documented using dedicated scales (VAS), as well as the pain and activities of daily life parts of the Lequesne scale and the global Lequesne Functional Index.

In contrast to prior research, our study did not yield evidence supporting the use of the procaine complex for alleviating depression [45,46]. However, consistent with the existing literature, we observed a significant role for physical therapy in managing depression symptoms [47,48,49]. In addition to oral NSAIDs or neuropathic medications, our study examined various interventions’ potential to enhance patient well-being by reducing pain, alleviating distress, or enhancing mobility. Unlike prior inquiries, periarticular injection demonstrated few adverse side effects and led to increased functional activities.

The study and meta-analysis published in the Journal of Post-Acute and Long-Term Care Medicine (JAMDA) shows a strong correlation between chronic pain and sarcopenia in elderly individuals. According to this research, patients with pain were 11% more likely to develop sarcopenia than those without pain, and sarcopenia was identified as independently associated with chronic pain [50,51]. There was no statistically significant difference in IADL performance between people with coxarthrosis and gonarthrosis (*p* = 0.3532). When comparing our study to that of Oliveria and colleagues’ [52] examining age- and sex-standardized incidence rates of hip and knee symptomatic pain, the gender-specific differences in the percentage of people with gonarthrosis or coxarthrosis were not statistically significant (χ^2^ = 0.005; df = 1; *p* = 0.940). Various confounding factors such as age, weight, level of physical activity, sample size, and individual variability may contribute to these findings.

In a meta-analysis on the efficacy of intra-articular hyaluronic acid for the treatment of gonarthrosis, Arrich et al. found a mean difference in VAS scores of −8.7 mm between the hyaluronic acid treatment group and the control group when all trial results were pooled [53]. Both hyaluronic acid treatment and procaine treatment resulted in a statistically significant improvement in pain reduction, as indicated by *p*-values below the conventional threshold of 0.05 when comparing treatment group data. Based on the negative mean differences, it appears that both therapies successfully relieve pain.

One may argue that procaine, the first injectable local anesthetic, was the precursor to all contemporary local anesthetics. Procaine, a molecule consisting of a lipophilic aromatic head, a hydrophobic terminal amine tail, and a hydrophilic aromatic head joined to the aromatic acid by an ester link, is created when an aromatic acid (para-aminobenzoic acid) and an amino alcohol mix [18]. The evidence for the medical use of the procaine complex (GH3) is, for the most part, based on results of observational studies and case reports in which it has been used in the treatment of several diseases with good effectiveness.

**Figure 3:**
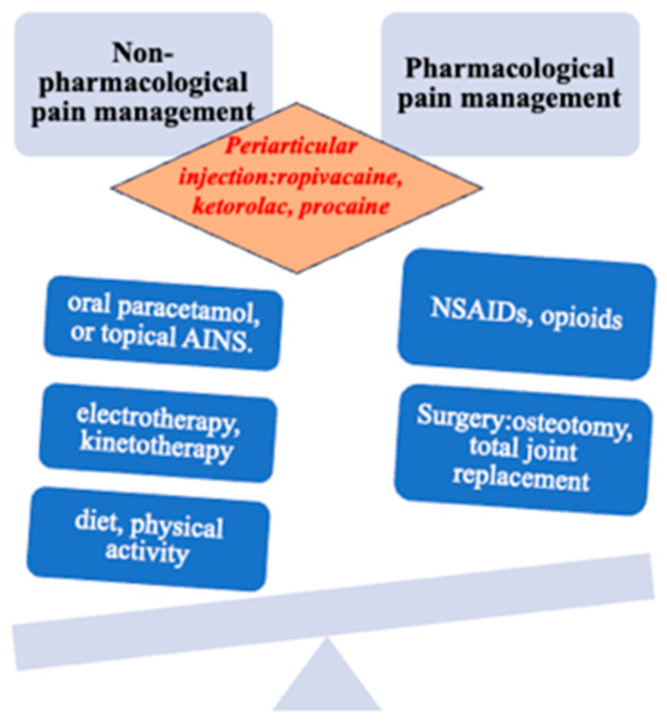
New treatment option for pain.

For example, in a recent meta-analysis, 60% of trials assessed the effect of drug treatment, and 26% evaluated surgical procedures. The lack of studies evaluating rehabilitation techniques, including bracing and other self-management techniques, has been labeled “research agenda bias” [54] and is, in part, a consequence of lucrative opportunities for drug development. The toxicity and adverse event profile of the most commonly used existing treatments (such as NSAIDs, cyclo-oxygenase-2 (COX 2) inhibitors, and total joint replacement) are unfavorably compared with conservative interventions such as exercise, weight loss, braces, and orthotics [55,56]. Some management guidelines are based on evidence from trials and expert consensus (see additional educational resources). The recommended hierarchy of management should consist of nonpharmacological treatments first, then drugs, and then, if necessary, surgery (Figure 3). Too often the first step is forgotten or not emphasized sufficiently, to the patient’s detriment [56], including young people [57].

Periarticular multimodal analgesia (PMA) injections are used to improve postoperative pain management by decreasing local inflammatory responses after total knee arthroplasty; nevertheless, the technique of administering the medicine plays a crucial role in their effectiveness. Due to its innervation, the periosteum of the tibia and femur is the main target of the injection around the knee [58]. Numerous research studies have looked into the benefits of periarticular drug infiltration. Jiang et al. found that the infiltration of analgesics for pain control provides better pain reduction than intravenous analgesia alone [59]. The potential for alleviating pain in oncological conditions should be examined and researched in the future [60]. The effectiveness of this alternative pain management technique with the procaine complex in elderly patients’ rehabilitation requires further research.

### 4.3. Limitations and Novelties of the Study

#### 4.3.1. Bias in the Selection Process

Randomization is a critical component in clinical trials to ensure the unbiased allocation of participants to different treatment groups. However, when it comes to elderly patients, particularly those with osteoarthritis and chronic pain, certain biases may still influence the randomization process. Elderly patients often have multiple comorbidities, which may lead to their exclusion from clinical trials. This selection bias can result in a study population that is not fully representative of the general elderly population suffering from osteoarthritis, potentially limiting the generalizability of the study’s findings. Older adults with higher levels of pain or functional limitations may be more inclined to participate in studies seeking new pain relief interventions, which can skew the sample towards those with more severe symptoms. Conversely, those with milder symptoms may opt out, leading to an overestimation of treatment effects.

#### 4.3.2. Study Limitations

The low number of subjects and the short study period limit the analysis. No personality traits were analyzed, with a relevant variable missing. Considering that depression is linked to personality, individual characteristics should be considered in the future.

However, the study’s evidential strength in establishing a causal relationship is comparatively less robust than other study methodologies. Nonetheless, it can be used as a documented starting point for further research using more rigorous scientific methods. A weakness of the study is the absence of a placebo group. A proper sample size estimation was regrettably not conducted before the commencement of this investigation. The participant count was established based on the accessible patient population that satisfied the inclusion criteria during the study period. We recognize that the absence of a pre-study sample size calculation constitutes a restriction of our research. Future research should focus on using bigger sample sizes to improve statistical power; prolonging the length to evaluate long-term benefits; and examining personality traits and psychological aspects that may affect treatment outcomes. Moreover, the systematic collection of adverse events and comprehensive reporting on intervention protocols would enhance the validity and application of the findings.

A disadvantage of this study is the absence of monitoring or restriction of participants engaging in concurrent interventions during the study period. We advised participants to avoid additional treatments related to the study outcomes, but we did not systematically verify adherence to this guidance. This may present potential confounding variables that should be addressed in subsequent studies.

#### 4.3.3. Safety and Tolerability

Periarticular procaine complex treatment has been shown to be safe for use. Adverse events are rare and transitory pain in the knee at the moment of procaine puncture. In the present study, adverse effects were not collected, but they were recorded in three patients (dizziness and orthostatic hypotension) and included only puncture accidents. Treatment compliance was 98.88%, with two drop-outs in the procaine group due to allergies.

#### 4.3.4. The Strength of the Study

This study focuses on osteoarthritis and its effects on pain management in older people, a demographic frequently under-represented in clinical research. The study investigates novel therapies, including the inaugural application of procaine in periarticular injections for geriatric patients in Romania, perhaps paving the way for new pain management methodologies. The study concentrates on older individuals with osteoarthritis, yielding data that are pertinent to practical clinical environments, with the objective of enhancing pain treatment and functional outcomes in this demographic. The study’s findings may improve clinical guidelines, improving treatment regimens and patient outcomes in treating chronic pain from osteoarthritis in elderly individuals. Their polypathology, inability to ingest oral drugs, and diminished pain threshold mentally hinder their compliance with physicians’ recommended physical workouts.

### 4.4. General Hypotheses and Clinical Implications

The management of osteoarthritis necessitates an individualized approach and often involves a combination of treatment modalities. Adjustments to the treatment plan should be made based on the patient’s response. Unfortunately, the majority of tested and utilized treatments revolve around pharmaceutical interventions, surgical procedures, or a combination of both. Immersive virtual reality is recognized in the therapeutic domain for its significant potential to enhance compliance, serving as a tool that facilitates behavioral modification. The findings of the study by Ramirez et al. indicate that an immersive virtual reality fitness program yields moderate-to-strenuous physical activity levels, with no significant differences observed across sexes [61].

Pain therapy for other conditions, such as migraine or myofascial pelvic pain, also involves a preventative strategy [62]. The predominant preventative strategy for migraines involves pharmaceutical and non-pharmacological desensitization therapies. Onabotulinumtoxin-A is a prevalent pharmaceutical treatment for chronic migraine, although physical therapy (PT) is typically regarded as an effective non-pharmacological alternative [63]. Deodato et al. examined whether a combination of pharmacological and non-pharmacological therapies may attain a superior desensitization rate in individuals with chronic migraine compared to typical monotherapy treatments. Our research utilizes a comparable methodology in elderly individuals experiencing osteoarticular pain, revealing beneficial outcomes when combined with physical exercise [63]. Pain linked to injured peripheral nerves frequently results from impaired signaling; however, successful nerve regeneration can alleviate this pain by reinstating appropriate communication between damaged fibers and their target tissues. Diverse pain modalities necessitate intricate management strategies [64].

Given the aging population and significant epidemiological developments, the rehabilitation health plan, which has consistently aimed to enhance functioning and reduce impairment, will emerge as a crucial health strategy. The growing significance of the rehabilitative approach in the health system supports the case for incorporating functioning data as a crucial element in national health information systems [65].

## 5. Conclusions

The use of a procaine-based drug to mitigate discomfort and improve movement in elderly individuals may represent a novel approach for treating frail seniors with arthrosis. These preliminary results indicate that the elderly group, aged 70–74 years, and those with osteoarthritis of the POA can primarily benefit from this treatment. Given that physical therapy appears to improve depression, we can develop and test a complex care plan in the future that combines both procaine administration and physical therapy.

A periarticular infiltration with the procaine complex, as a conservative complementary pain therapy, can be beneficial at the start of a rehabilitation program in frail elderly patients with a high VAS value.

## Figures and Tables

**Table 1:** Baseline characteristics of the study group.

Category	Values
Age (mean)	70.62 ± 8.87 years
Sex	F/M = 154 (87%) vs. 23 (13%)
Residence	U/R = 121 (68.75%) vs. 55 (31.25%)
Diagnosis	
POA	61 (34.46%)
SOA	26 (14.69%)
SPOA	73 (42.24%)
HD	14 (7.91%)
Osteoporosis	2 (1.13%)
Trauma	1 (0.56%)

POA = gonarthrosis, coxarthrosis, and osteoarthritis of the peripheral joints; SOA = spine osteoarthritis; SPOA = both central (spine) and peripheral joints affected by osteoarthritis; HD = spinal disc herniation and sciatica.

**Table 2:** Longitudinal comparison of the mean values for the analyzed parameters between examinations on day 1 (T0) and T1, as well as the improvement.

Index	Time	Experimental Group (Mean ± SD)	Control Group (Mean ± SD)	*t*-Test (df *)	Significance (*p*)
VAS	T0	6.4 ± 2.01	3.03 ± 2.20		
	T1	3.86 ± 2.07	2.39 ± 1.83		
VAS T0–T1	T0–T1	2.54 ± 1.37	0.63 ± 0.88	11.28 (171.07)	<0.001
P/D	T0	4.66 ± 2.05	3.33 ± 2.16		
	T1	2.72 ± 1.75	2.04 ± 1.38		
P/D T0–T1	T0–T1	1.95 ± 1.36	1.29 ± 1.15	3.46 (172.59)	=0.001
MDW	T0	3.22 ± 2.04	2.71 ± 2.32		
	T1	2.44 ± 1.7	2.09 ± 2		
MDW T0–T1	T0–T1	0.78 ± 0.69	0.64 ± 0.56	1.41 (173.92)	=0.159 (NS)
ADL	T0	3.88 ± 1.91	3.79 ± 2.46		
	T1	3.13 ± 1.8	3.43 ± 2.18		
ADL T0–T1	T0–T1	0.75 ± 0.58	0.31 ± 0.64	4.68 (152.46)	<0.001
LAI	T0	11.78 ± 5.19	9.72 ± 5.88		
	T1	8.43 ± 4.38	7.53 ± 4.68		
LAI T0–T1	T0–T1	3.36 ± 1.91	2.20 ± 1.61	4.36 (172.68)	<0.001
GDS	T0	4.17 ± 3.22	2.83 ± 2.32		
	T1	3.99 ± 3.5	2.16 ± 1.69		
GDS T0–T1	T0–T1	0.18 ± 2.22	0.67 ± 1.10	−1.93 (153.90)	=0.055 (NS)

* df = degree of freedom. (NS = non significant).

**Table 3:** Parameter improvement at discharge for the different diagnoses.

Index	Diagnosis	Mean ± SD	F-Test	Significance (*p*)
VAS T0-T1	POA	2.25 ± 1.48	3.88	=0.002
	SOA	1.73 ± 1.56		
	SPOA	1.26 ± 1.40		
	HD	2.07 ± 1.44		
	Osteoporosis	0		
	Trauma	1		
P/D T0-T1	POA	1.91 ± 1.36	1.11	=0.358 (NS)
	SOA	1.77 ± 1.45		
	SPOA	1.40 ± 1.23		
	HD	1.79 ± 1.25		
	Osteoporosis	1.5 ± 0.71		
	Trauma	2		
MDW T0-T1	POA	0.74 ± 0.70	0.70	=0.621 (NS)
	SOA	0.75 ± 0.65		
	SPOA	0.67 ± 0.62		
	HD	0.93 ± 0.27		
	Osteoporosis	0.50 ± 0.71		
	Trauma	0		
ADL T0-T1	POA	0.62 ± 0.67	0.20	=0.964 (NS)
	SOA	0.56 ± 0.45		
	SPOA	0.52 ± 0.73		
	HD	0.50 ± 0.39		
	Osteoporosis	0.50 ± 0.71		
	Trauma	0.50		
LAI T0-T1	POA	3.16 ± 1.89	0.84	=0.524 (NS)
	SOA	2.96 ± 1.95		
	SPOA	2.54 ± 1.89		
	HD	3.14 ± 1.60		
	Osteoporosis	2.50 ± 2.12		
	Trauma	2.50		
GDS T0-T1	POA	0.38 ± 1.07	0.68	=0.636 (NS)
	SOA	0.23 ± 0.76		
	SPOA	0.33 ± 2.54		
	HD	0.71 ± 1.64		
	Osteoporosis	2.50 ± 0.71		
	Trauma	1.00		

(NS = non significant).

**Table 4:** Parameter improvement at discharge by age category.

Index	Category	Mean ± SD	F-Test	Significance (*p*)
VAS T0-T1	Adult	1.65 ± 1.61	3.39	=0.019
	Elderly	2.24 ± 1.66		
	Very old	1.31 ± 1.12		
	Oldest old	1.64 ± 1.29		
P/D T0-T1	Adult	1.55 ± 1.34	0.93	=0.428 (NS)
	Elderly	1.90 ± 1.39		
	Very old	1.54 ± 1.28		
	Oldest old	1.82 ± 0.87		
MDW T0-T1	Adult	0.59 ± 0.61	2.37	=0.073 (NS)
	Elderly	0.79 ± 0.66		
	Very old	0.73 ± 0.63		
	Oldest old	1.09 ± 0.54		
ADL T0-T1	Adult	0.48 ± 0.65	0.69	=0.561 (NS)
	Elderly	0.66 ± 0.85		
	Very old	0.55 ± 0.39		
	Oldest old	0.59 ± 0.49		
LAI T0-T1	Adult	2.52 ± 2.00	1.46	=0.226 (NS)
	Elderly	3.17 ± 1.78		
	Very old	2.85 ± 1.81		
	Oldest old	3.41 ± 1.71		
GDS T0-T1	Adult	0.23 ± 2.76	0.41	=0.749 (NS)
	Elderly	0.35 ± 1.02		
	Very old	0.55 ± 0.97		
	Oldest old	0.73 ± 1.10		

(NS = not significant).

**Table 5:** Distribution of “Up and Go” test scores.

“Up and Go” Test	Mean ± SD	Significance (*p*)
Gonarthrosis	13.13 ± 5.601	0.0305
Coxarthrosis	15.7 ± 5.166	

## Data Availability

Data available on request from the authors.

## Abbreviations

ADL	Activities of Daily Living
CG	Control Group
CGA	Complex Geriatric Assessment
COVID-19	Coronavirus Disease 2019
COX-2	Cyclo-oxygenase-2
CpG	Cytosine–Guanine
DEAE	Diethylaminoethanolic Acid
DNA	Deoxyribonucleic Acid
EG	Experimental Group
ESR	Erythrocyte Sedimentation Rate
GDS	Geriatric Depression Scale
HD	Spinal Disc Herniation
IADL	Instrumental ADL
JAMDA	Journal of Post-Acute and Long-Term Care Medicine
MAO	Monoamine Oxidase
MMSE	Mini Mental State Examination
NS	Not Statistically Significant
NSAIDs	Nonsteroidal Anti-Inflammatory Drugs
OA	Osteoarthritis
PAB	Para-Aminobenzoic acid
PMA	Periarticular Multimodal Analgesia
POA	Osteoarthritis of the Peripheral Joints
PT	Physical Therapy
QoL	Quality of Life
SD	Standard Deviation
SOA	Spine Osteoarthritis
SPOA	Osteoarthritis of the Central (spine) and Peripheral joints
VAS	Visual Analog Scale
UK	United Kingdom
US	United States
